# Sociocultural Determinants of Nutritional Status Among Children Under Five Years of Age: An Ethnographic Study From Gujarat

**DOI:** 10.7759/cureus.27377

**Published:** 2022-07-27

**Authors:** Tanveer Umallawala, Tapasvi Puwar, Apurvakumar Pandya, Priya Bhavsar, Manoj S Patil, Somen Saha

**Affiliations:** 1 Epidemiology, Indian Institute of Public Health, Gandhinagar, IND; 2 Public Health, Indian Institute of Public Health, Gandhinagar, IND; 3 Parul Institute of Public Health, Parul University, Vadodara, IND; 4 School of Epidemiology and Public Health, Jawaharlal Nehru Medical College, Datta Meghe Institute of Medical Sciences, Wardha, IND

**Keywords:** social and community determinants, gujarat, devbhumi dwarka, ethnography, severe acute malnutrition (sam)

## Abstract

Background

The magnitude of child malnutrition, including severe child malnutrition, is high in India, and Gujarat has a higher prevalence of child malnutrition. Prior studies have employed anthropometric measures to identify the prevalence and associated factors of children’s undernutrition. The present study explored community-level determinants of malnutrition among malnourished and well-nourished children in Devbhumi Dwarka district of Gujarat State, India.

Methods

A qualitative research employing focused ethnographic methodologies was used. In-depth observations of 60 families in a home food environment were carried out. Each child was observed at their respective homes for three consecutive days. Data were analyzed using thematic analysis techniques.

Results

The study revealed that lack of knowledge on malnutrition, inadequate feeding practices, poor socioeconomic status, insufficient hygiene and sanitation practices, lack of food variety, use of health facilities, and birth complications were the major community-based determinants of malnutrition.

Conclusion

The study identified community-level determinants of malnutrition among children under five years in the Devbhumi Dwarka district. To tackle the immediate and underlying causes of malnutrition, interventions are urgently needed to create community awareness about malnutrition as a disease and optimal infant and young child feeding (IYCF) practices using behavior change communication strategies.

## Introduction

Malnutrition is a public health concern across the globe. According to the WHO, malnutrition refers to deficiencies, excesses, or imbalances in a person’s intake of energy and/or nutrients [1]. According to the National Family Health Survey (NFHS-4), 35.7% of children below five years are underweight, 38.4% are stunted, and 21% are wasted, whereas in Gujarat, 39% of children below five years are underweight, 39% are stunted, and 26% are wasted [2]. Gujarat has the highest prevalence of malnutrition in India. According to the Global Nutrition Report 2018 [3], India is home to 46.6 million stunted children, and 69% of all under-five child mortality in India is attributable to undernutrition [4]. Undernutrition puts children at greater risk of common infections, and the severity of such infections delays recovery or increases the risk of dying [5].

Low socioeconomic status, food insecurity, mother’s age at birth, birth interval, large family size, illiteracy in the family, maternal autonomy to make decisions, and poor hygiene and sanitation are factors responsible for high levels of child malnutrition in developing countries, including India [6-11]. A study conducted in Uttar Pradesh, India, revealed that prelacteal feeding, which is a barrier to exclusive breastfeeding, inadequate complementary feeding practices, and lack of knowledge about feeding practice in caregivers were associated with a decline in child’s growth [9]. The hardest hit, according to UNICEF, can be a lack of essential healthcare treatment, lack of education of the mother, insufficient practice of exclusive breastfeeding, and poverty [12]. A conceptual frame on the determinants of child’s undernutrition is well presented by UNICEF [13], which highlights a comprehensive understanding of multiple factors in social and community contexts causing malnutrition that operates at the “immediate,” “underlying,” and ”basic” level. All these factors together contribute to a vicious cycle of inadequate dietary intake and frequent diseases resulting in malnutrition in young infants. Previous studies in Gujarat had explored the prevalence and associated contributing factors of a child’s undernutrition employing anthropometric measures [14-17]. The current study explored underlying community determinants of undernutrition among children under five years of age in the Devbhumi Dwarka district of Gujarat. The main purpose of the study was to understand the dietary practice, infant and young child feeding (IYCF) practices, food hygiene, food storage in the families, and utilization of health and nutrition services among malnourished and well-nourished children.

## Materials and methods

Study setting

The study was conducted in the Devbhumi Dwarka district, which is located on the southern coast of the Gulf of Kutch in Gujarat. The district is divided into four blocks: Dwarka, Kalyanpur, Khambhaliya, and Bhanvad. The district has 235 km of coastline; hence, a few villages of Dwarka and Khambhaliya are situated near sea coasts. The study primarily covered the rural areas, including villages situated near the coastal area. Agriculture, animal husbandry, fishing, and petty trading were the major occupations. Most families were farmers, laborers, and petty traders. The trading activities ranged from selling food items, stationery, household utensils, garage, and tailoring shop. The majority of the population, including women, chew tobacco and snuff, which is locally called “faki.” Most villages were characterized by people from Hindu and Muslim religions and from different castes living together but on different streets. Communal segregation was common with houses predominantly segregated by homogenous caste and community. The communities that inhabited the selected villages were Kolis, Kathis, Rabaris, Bharwad, and Lohana, and artisans such as tailors, potters, carpenters, and barbers.

Study design

A focused ethnography approach was employed. It deals with a distinct problem in a specific context and is conducted within a subcultural group with an assumption that the participants share a cultural perspective. The researcher focuses on the participants’ common behaviors and shared experiences [18-20]. In the present study, children of selected families with malnourished (MN or SAM) and well-nourished (WN or Non-SAM) were observed for three consecutive days. The observation was recorded using notes. The observation checklist included themes such as food preparation process, IYCF practices, cooking practices, practices on food hygiene and food storage, types of food given to children, dietary diversity, animal care, home hygiene practices, child’s personal hygiene practices, utilization of services related to nutrition, and treatment-seeking behaviors. Participant observation for three days was scheduled as presented in Table 1.

**Table 1:** Participant Observation Schedule

Day	Key areas of participant observation
First	Building rapport with a family to figure out basic habits of children and family members
Second	Observation of child-caretaker interaction, conversation with mother/caretaker, and participation in activities with mothers such as accompanying mother to the market and helping her in cooking and other household chores
Third	

Participant observation was carried out by a trained researcher. They visited the participant’s homes daily. Conversation with mothers/caregivers of children was a strategy adapted by the researchers to understand behavior, knowledge, and cultural belief related to the nutritional requirement of the child, while participation in household activities focused on understanding the utilization of Integrated Child Development Services (ICDS), IYCF practices, and treatment-seeking behaviors of caretakers.

Data collection

**Figure 1:**
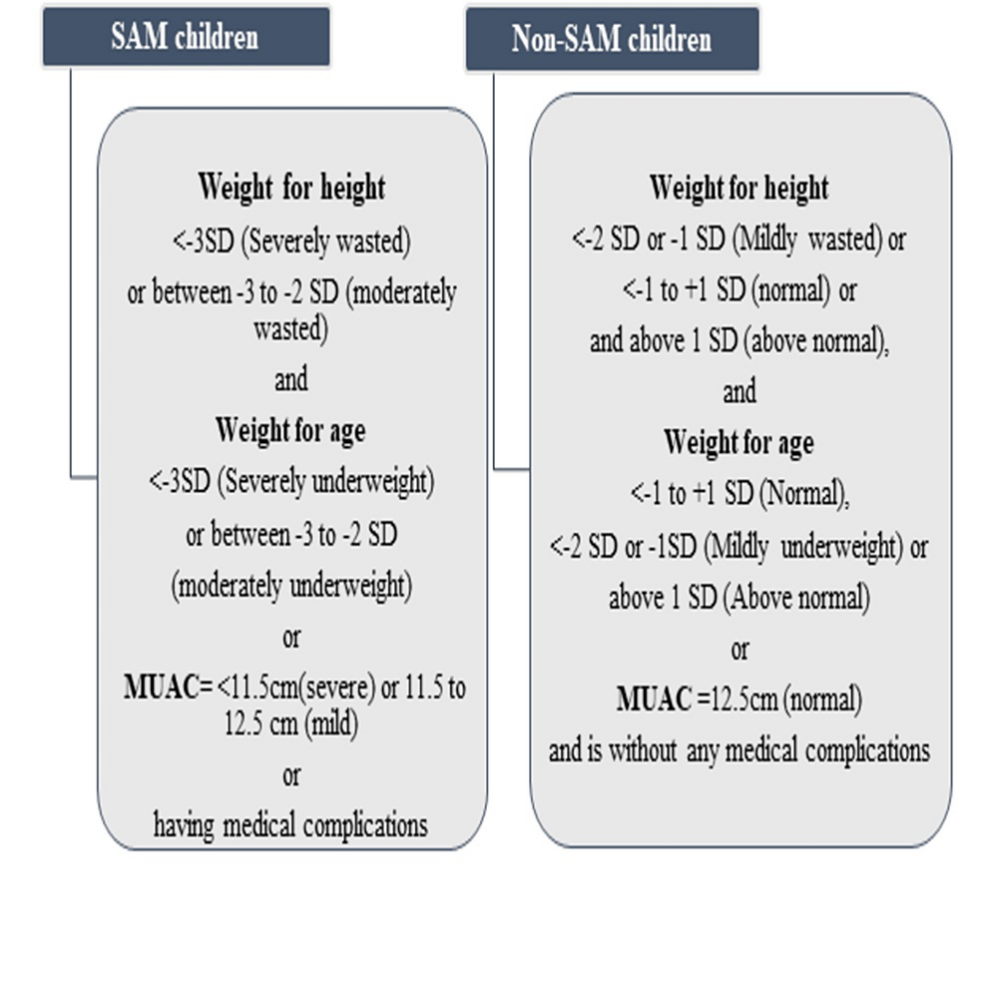
Selection Criteria of the Study Population

Data on malnourished children were taken from records maintained by the local health department. Anthropometric and clinical assessments of MN children were conducted manually, and those children who were actually notified as MN were selected as the population for the study. The criteria for the selection of MN and WN children are presented in Figure 1. A total of 30 MN children and 30 WN children under five years of age were purposively selected using the following inclusion criteria: both MN and WN children live in the same family or nearby households in a similar geographical location of the village, the respondents of children were mothers or caregiver, and consent for participation in the study was provided.

The observation checklist was developed in English and then back-translated into Gujarati. The tool was reviewed by public health and nutrition experts, pilot tested for validity, and modified accordingly. During the home visits, observations were noted on recording devices instead of paper to avoid distracting the family.

Data were analyzed using thematic analytic techniques. Observations were recorded in a local language, ensuring that investigators captured not only the spoken words but also the setting, context, body language, and general interactions within the family between mothers and children dyad [19]. Both handwritten notes and digitally written data on the mobile phones were used for transcription. Transcripts were translated into English. The codebook was developed in an Excel sheet to manage evolving coding schemes; descriptions of the content relevant to codes and examples from the data were used as references. Coding was done manually by reading and re-reading transcripts to identify patterns and categorized codes into themes. Frequencies of emerging codes and their categories were counted. Frequencies, co-occurring codes, and reflective memos were used to describe themes. In the end, based on emerging concepts, pathways to malnutrition were identified.

Formal approval was obtained from the state and district authority. The study was approved by the Institutional Ethics Committee of the Indian Institute of Public Health, Gandhinagar (IEC/IRB approval number 14/2019-20) on May 29, 2020.

## Results

Basic characteristics of the study participants

A total of 25 children in the category of severe acute malnutrition (-3 or <-3 SD) and five moderate malnutrition (-3 to -2 SD) were grouped as malnourished children (group I). The detailed nutritional characteristics of the study participants are depicted in Table 2.

**Table 2:** Characteristics of the Study Participants

Variables	Group I (malnourished children) (N=30)	Group II (well-nourished children) (N=30)
Anthropometric measurements		
Severe (-3 or	21	0
Moderate (-3 to -2 SD)	9	0
Normal (-1 to +1 SD)	0	30
Mid-upper arm circumference		
<11.5	5	0
≥11.5	25	30
Birthweight		
Less than 2.5 kg	18	1
More than 2.5 kg	10	25
No record found	2	4
Medical complications	2	0
Birth order		
1	11	12
2	12	13
3	6	2
4 or more	1	2
Sociodemographic characteristics		
Gender		
Males	18	12
Females	12	18
Age group		
Birth to three years	17	20
3-5 years	13	10
Type of family		
Joint	19	16
Nuclear	11	14
Caste		
Schedule class/schedule tribe	3	3
Other backward class	17	17
General	10	10
Religion		
Hinduism	25	26
Muslim	5	4
Caretaker		
Mother	21	24
Others	9	6

About 18 children in group I had a birth weight below 2.5 kg, and the other two had medical complications. Similarly, in group II (well-nourished children), six fell into the mild malnutrition category (-2 to -1 SD), whereas 24 of them were normal (-1 to +1 SD). Most well-nourished children (n=25) had a birth weight of more than 2.5 kg except one. In terms of sociodemographic characteristics, the gender of children was equally distributed in both groups (18 boys and 12 girls in group I, and 12 boys and 18 girls in group II). Joint families were predominant; 19 children from group I and 16 in group II lived in joint families. The detailed sociodemographic characteristics of the study participants can be seen in Table 2.

The major seven themes that emerged from the data were socioeconomic status, behavior and ethnicity, parity and previous birth history, feeding practices, home hygiene and sanitation, cultural dietary habits, and utilization of health and nutrition services.

Socioeconomic status

Some families of malnourished children (n=20) were daily wagers or farm laborers whose socioeconomic conditions were weaker compared to others who were farmers (n=6), teachers (n=1), and petty traders (n=3). Most mothers (n=22) were housewives, while eight were laborers. Similarly, families of well-nourished children were laborers (n=14), farmers (n=6), doing a job (n=5), and petty traders (n=5). Except for five mothers, all were housewives. Comparatively, malnourished children families were socioeconomically weaker than that of well-nourished children families (20 versus 14 daily wagers in groups I and II, respectively); eight and five mothers were laborers in groups I and II, respectively. For those who were daily wager/laborers in both groups, their food consumption depended on their daily wage. Hence, their capacity to purchase food was limited, which precluded them from consuming nutrient-rich food. The staple diet among these communities across both groups was bajra rotla (millet bread), rice, dal (yellow split pigeon pea curry), and potato. Further, food purchase and the consumption pattern across both groups were similar, except among daily wagers/laborers. Few families (four in group I and six in group II) were purchasing fruits for children. One family in group II was well-off, but the child’s dietary practices did not include nutrient-rich leafy vegetables and fruits as families did not perceive the importance of leafy vegetables and fruits and because of the inability to inculcate healthy dietary habits in children. One participant said, “My child does not eat healthy food despite what we offer. She eats potato chips and fries … Every day, (she) needs candy.”

Behavior and ethnicity

Most of the families (n=35) were joint families, and others (n=25) stayed on the farm, which was approximately 3-4 km from their parent’s home in the village. Most families in both groups were patriarchal, dominated by male members, and the involvement of fathers in childcare was minimal. Among families with malnourished children, most children (n=26) visited Anganwadi centers (AWCs). Three parents were not sending their children to AWCs as they belonged to a lower caste; one family was better-off and hence did not consider AWCs and instead sent their child to a private preschool.

In some cases (n=9), caretakers of children were grandmothers, elder siblings, or neighbors, and they did not attend to children in terms of preparing food for children, maintaining hygiene in the house, and handwashing before giving food to children. In contrast, among well-nourished children families, mothers took care of their children with the support of other family members, especially mothers-in-law. Most mothers did not go to work until the child was six to seven months. Whenever they had to go to work, children were cared for by grandmothers and elder children who took care of the children by offering timely fresh cooked food and playful interactions with young children. For example, in one household where the mother was a laborer who started working two months after delivery, the mother-in-law used to take the child to her mother for breastfeeding whenever the child demanded: “I had to go work daily and leave my child with my mother-in-law who used to take care of her. Whenever the child cries, she used to bring the child to me to breastfeed her ….”

Such care was lacking in the group with malnourished children, which might have impacted the child’s nutritional outcomes. However, families of well-nourished children (n=18) did not utilize AWC services compared to malnourished children families (n=26). Casteism strongly emerged as a barrier to accessing services. Families from the upper caste did not allow auxiliary nurse midwives (ANM) and accredited social health activists (ASHA) from the lower caste to visit their houses. Childhood illnesses were not attended to seriously due to superstition in both groups. For example, at one household, when the investigator asked the mother to see a doctor as a child seem to have a fever and looked weak, the mother responded, “She (the child) was having a fever for a few days; we went to Bhuva (faith healer) who told us the impact of bad spirit (bhut ni chhaya) on her and gave a holy thread (dhaga) to protect her. She will recover soon.” Such superstitions and cultural beliefs prevent parents from treating childhood illnesses timely with modern medicines.

Despite the fact that most mothers (n=45) across both groups were housewives, they were unable to take care of children in terms of providing freshly cooked food and handwashing practices during critical periods such as before feeding the child, cooking, and after cleaning the home due to lack of awareness on correct knowledge on food, hygiene, and childcare practices. Working mothers (n=15) had the most difficulties in exclusive breastfeeding, weaning practices, and complementary feeding. However, among well-nourished children, family support in childcare was an important factor.

Parity and previous birth history

The history of multipara was seen in the majority of the mother of MN children, and there was no birth spacing between the two pregnancies. In families with WN children, women with adequate birth space were maintained. When asked about the history of their children, the majority of the mothers from families with MN children had low birth weight (LBW), medical complications, and childhood illnesses. In some mothers of MN children (n=5), previous children below five years were well-nourished. The number of frequent pregnancies might be the cause of the malnourishment of the recent child. Furthermore, childhood illnesses such as fever, common cold, cough, convulsions, and irritability in the behaviors of MN children were not attended to by the mother or other family members.

In mothers of MN children who had a history of parity or diminished quality or quantity of breastmilk or medical complications (such as premature birth), children were fed with milk powder, cow milk, or goat milk, which precluded providing colostrum feeding and exclusive breastfeeding. Of the total, 11 mothers of MN children could initiate breastfeeding within an hour compared to mothers of WN children (n=21).

Families with WN children were characterized by the absence of medical complications during birth, adequate birth weight and birth spacing, and immediate treatment of childhood illnesses. Few children (n=4) with low birth weight were provided milk powder as prescribed by private practitioners. Children in these families were playful, and family members interacted with children when the mother was occupied with household chores.

Feeding practices

Mothers from both MN and WN children reported giving prelacteal feeding. Mothers of WN children believed that prelacteal feeds “boost immunity,” “facilitate digestion,” and “remove infections within the body.” Mothers of MN children also informed that giving these feeds was the norm. Prelacteal feeds that were commonly fed were honey, water, jaggery water “ghutti” (tonic made from herbs, additives, and honey), goat milk, or cow milk. These were given frequently, sometimes every hour, as advised by mother-in-law, neighbor, or relatives. Few children (n=4) with low birthweight were provided milk powder as prescribed by private practitioners.

More than half of MN children (n=16) were not provided colostrum feeding due to beliefs such as “not good for the child” and “advice from a doctor to discard colostrum.” Although prelacteal feeds were given, many mothers of MN children (n=19) and most mothers of WN children (n=21) initiated breastfeeding within an hour after birth, but MN children (n=19) were not exclusively breastfed. In a few cases of MN children, milk powder was provided due to complications such as LBW at the hospital or diminished production of breastmilk; hence, breastfeeding was delayed. Partial breastfeeding was practiced by both groups of mothers since birth. The use of animal milk (cow or goat milk) diluted with water was the most common practice. The reasons for using animal milk for feeding were an insufficient quantity of milk produced, the mother’s illness, and the child being not able to suck breastmilk due to illness or weakness since birth.

Families with WN children were characterized by exclusive breastfeeding and timely and adequate complementary feeding. Children in these families were playful, and family members were interacting with children when the mother was occupied with household chores.

Home hygiene and sanitation

For mothers of both groups, handwashing practices were insufficient; 17 MN children and 17 WN children did not practice handwashing, especially before cooking, after cleaning, and providing food to children. However, the utensils used for cooking and serving food used by mothers of MN children were not cleaned before their use. Furthermore, food was usually cooked in the morning, around 8:00 AM to 8:30 AM, and the same food was served during the day. Food was stored outside the kitchen, which was not cleaned regularly. Among nine families of MN children, a food storage facility was unavailable in the house. General cleanliness was not maintained in houses, and most houses had no toilet facility at their home. Thus, open defecation was practiced either outside the house or in the backyard. Areas where children defecated were not usually cleaned, and children were fed in the same areas.

As mentioned earlier, more than half of the mothers of WN children (n=17) did not practice handwashing. Thirteen mothers of WN children washed their hands before and after critical activities (cooking, cleaning the house, using the toilet, and offering food to children) but without soap. Nevertheless, unlike the mothers of MN children, in this case, utensils were cleaned with water and ash, and food storage practices were hygienic. Few families (n=11) stored cooked food in a refrigerator. Although the food was cooked in the morning and families used to eat the same food at noon, the mothers cooked fresh food when the child demanded.

Inadequate dietary intake was observed in both groups. The foods frequently used for feeding MN children (n=21) and WN children (n=19) were either ready-made junk food such as biscuits, fries (bhungada), fried potato chips, bread, or occasionally homemade recipes (such as porridge, rice starch, or pulse water) or recipes based on premixed packets of take-home ration (THR) provided by the government for infants and young children. Infrequently, fruits, vegetables, rice, pulses, and cereals were also used. Commercially available infant cereal in both groups was not used, but few mothers in both groups used baby powders as prescribed by private practitioners.

When compared to WN children, there was less variety in foods given to MN children. Complimentary foods given to MN children included bajra rotlo (millet bread), tea, rice starch, or pulse water in the morning until noon. When the child demanded food from caretakers between morning snacks to lunchtime, ready-made junk foods were offered. MN infants consumed less quantity of complementary food (only one or two spoons) than WN children who consumed one standard bowl. Only a few MN infants/children consumed fruits and preparations from cereals (wheat and rice), pulses, and vegetables. In contrast, WN children had food diversity with cereals, pulses, rice, khichdi (pulse and rice cooked together with oil until semisolid), milk and buttermilk, fenugreek leaves, and mostly potatoes as a vegetable.

Cultural dietary habits

The use of premixed take-home ration (THR)-based recipes was discouraging among families of MN children. Most families of MN children objected to using premixed THR packets for children provided by the government. They believed that it induces vomiting. Mothers opined that children did not like the sweet taste of THR. In contrast, many families with WN children (n=19) fed their children with THR-based recipes. In both groups, milk was consumed; however, it was most commonly consumed in the form of tea.

Utilization of healthcare and nutrition facilities

During the conversation with the mothers of both groups, they stated that they were not advised to admit their children to the child malnutrition treatment center (CMTC). In most cases, when symptoms such as fever or common cold were observed, they took treatment from a private hospital. Some mothers of MN children were advised to admit their children to CMTC by Anganwadi workers (AWW) and ANM, but the mothers and family members perceived that there is no need to admit children to the CMTC if only the weight was less and if children were not having any diseases or symptoms that require urgent attention. One mother of a SAM child explained, “… Only weight is low … It means the (child) is not having any disease. Providing food will increase child’s weight.”

Malnutrition was not perceived as a health hazard. Mothers and caregivers were not aware that malnutrition, if unattended, may cause death or disease or impair the growth of their children. As a result, most families did not seek healthcare for it.

Some parents (n=3) wanted to treat their MN child, but there is no available CMTC facility in the district. One MN child was taken to the Nutrition Resource Centre (NRC) in Jamnagar (near the district as Devbhumi Dwarka did not have an NRC) but felt disappointed as the doctor had advised her not to admit the child to the center, suggesting that the child will be cured with medicines. A few days after medication, anxious parents consulted a private practitioner and were given baby powder for the children.

Most families with MN children (n=25) accessed ICDS services such as AWC, regular checkups, and THR. Many families encouraged children to visit AWCs for early childhood care and education. Participants who did not attend AWCs were informed that they were receiving premixed THR packets at home; however, as pointed out earlier, THR was not used to feed children as it was believed to cause vomiting among children. Moreover, THR packets were not as per the POSHAN ABHIYAN guidelines (10 packets per child/per month). Only one WN child in the age group of birth to two years received 10 packets of THR.

## Discussion

This qualitative home-based in-depth observation was conducted to identify the determinants of malnutrition at the community level by observing the behaviors and dietary practices of families of malnourished children and assessing the utilization of health facilities for the management and prevention of malnutrition. This study also reflected how the household environment of MN children differs from WN children.

Handwashing is an essential component of hygiene and sanitation practices. There was a lack of proper and timely handwashing practices by mothers and caretakers in both groups. However, food hygiene and general cleanliness among families of MN children were not maintained, which directly linked to the risk of infections or disease. A similar study in Nepal highlighted the material used for handwashing by mothers and the frequency of handwashing practice as a major causal factor for malnutrition in children [6].

Another major factor was the parity of mothers of MN children. Some illnesses and complications such as low birth weight and delayed initiation of breastfeeding were immediate causes of malnutrition, which may lead to death. A study showed that poor maternal health and malnutrition often result in the low birth weight of infants, which predisposes many of them to malnutrition early in life, and stunting was more observed in children with a minimum interval between two pregnancies [7].

Inadequate dietary intake was an immediate cause of malnutrition majorly observed in MN children compared to WN children. MN children were taking very little quantity of the food, and there was no variation in the food. For instance, almost all families feed their children with only milk, rotla, rice, etc., not giving green leafy vegetables, fruits, pulses, etc. Such limited food intake potentially leads to a deficiency of proteins, minerals, and other vitamins in children. Mothers of MN children were unaware of the diet to be given, while the diet of WN children was comparatively better, consuming nutritious food in adequate quantity. Various studies reported inadequate dietary intake as one of the key factors determining malnutrition [9,14-16].

The family’s socioeconomic status makes an appreciable effect on malnutrition in children, which adversely impacts the accessibility of nutritious food [17-21]. The majority of the people, especially laborers, were not able to attend to their child’s nutritional and developmental needs and had limited access to procure nutritious food. Many mothers were daily wagers, which might have resulted in insufficient care for themselves and for their children. In a nutshell, the primary factors of malnutrition that emerged from the study were low socioeconomic status, casteism, superstition, and inappropriate or faulty behavioral practices. Similar findings were reported in the study conducted in India [22] and Nairobi [23]. A review of NFHS-4 data by Singh, Srivastava, and Upadhyay [24] indicated a link between socioeconomic status and malnutrition.

The study shows that the utilization of health facilities by both groups was restricted. The primary reasons were the beliefs that “treatments are not required,” lack of knowledge about the treatment for malnutrition, and unavailability of CMTC service in the district. The majority of the family avoided visiting government health facilities during child illness as they preferred private service providers. Families were avoiding THR packets from AWC as they believed their children would be sick after consuming THR. Also, they avoided visiting AWC as well. Low uptake of health and nutrition services has been documented in various studies in India and other developing countries [14-18,25-30].

Inadequate infant and young child feeding practices may affect the nutritional status of children. In MN children, delayed initiation of breastfeeding, nonexclusive breastfeeding, and insufficient complementary feeding practices were observed. Davalgi et al. described in their study that only 39% of mothers initiated breastfeeding within half an hour of childbirth [31]. Another study from Bangladesh showed that exclusive breastfeeding among children under six months was significantly and positively associated with malnutrition [32]. Notably, the quality of complementary feeding was also a key determinant of positive nutritional status. A study conducted in 2012 showed that poor quality of complementary feeding was positively associated with a higher prevalence of stunting and malnutrition [33].

Increasing dietary diversity may effectively reduce the burden of stunting and chronic malnutrition among children under five years of age in the district. Interventions that address dietary diversity, such as unconditional cash transfers, community-based caregiver nutritional education [29], promotion of improved IYCF practices, [30] positive deviance health approach [31], and integrated community case management [32], should be considered in the district, in addition to improving knowledge on malnutrition and uptake of nutrition and healthcare services; discouraging junk food consumption and promoting the use of locally available nutrient-rich food for children through a positive deviance health approach; increasing access to family planning; improving water, sanitation, and hygiene (WASH) practices; and making available malnutrition treatment facility (CMTC and NRC) in the district.

## Conclusions

This study used an ethnographic approach of observing 30 families of malnourished and neighboring 30 families of well-nourished children to assess the determinants of malnutrition, including food preparation process, IYCF practices, cooking practices, practices on food hygiene and food storage, types of food given to children, dietary diversity, animal care, home hygiene practices, child’s hygiene practices, utilization of services related to nutrition, and treatment-seeking behavior. Some important patterns that emerged include birth weight, socioeconomic status, behavior and ethnicity, parity and previous birth history, feeding practices, home hygiene and sanitation, cultural dietary habits, and utilization of health and nutrition services. Malnourished children have lower birth weight, a history of multipara, nonexclusive breastfeeding, low socioeconomic status, insufficient family support, and poor hand hygiene practices, and their families believe in superstitions and myths, which hampered care-seeking; these determinants were considered the immediate and underlying causes of malnutrition.

The determinants of malnutrition can be addressed by improving community-based caregiver nutritional education, taking up health and nutrition services, avoiding junk foods, promoting locally available nutrient-rich foods for children through a positive deviance health approach, promoting IYCF practices, making available the malnutrition treatment facility in the district, improving water, sanitation, and hygiene (WASH) practices, etc. Behavioral change and communication interventions to create community awareness about malnutrition as a disease and to encourage optimal IYCF practices are needed to tackle the immediate and underlying causes of malnutrition. Lastly, mothers of well-nourished children’s involvement as peer counselors can be optimized for program effectiveness.
